# Empowerment-based support program for vulnerable populations living with diabetes, obesity or high blood pressure: a scoping review

**DOI:** 10.1186/s12889-022-14480-3

**Published:** 2022-11-09

**Authors:** Julia Eïd, Annabel Desgrées du Loû

**Affiliations:** 1 Centre Population et Développement (Ceped), Institut de Recherche pour le Développement (IRD), Université Paris Cité, Inserm ERL 1244, 45 Rue des Saints-Pères, 75006 Paris, France; 2 Association Ikambere, Saint-Denis, France

**Keywords:** Diabetes mellitus, Obesity, Hypertension, Patient education, Population health management, Vulnerable populations

## Abstract

**Background:**

The management of chronic diseases such as diabetes, obesity and high blood pressure is a major global health challenge, particularly among the most disadvantaged populations. Beyond the biomedical management of these diseases, comprehensive support that takes into account the peoples’ economic and social situation is fundamental. The objective of this scoping review is to create an inventory and an analysis of the different types of support for these chronic diseases among disadvantaged, immigrant or minority populations to contribute to a better definition and characterization of what should be global support for these vulnerable populations suffering from these diseases.

**Methods:**

A search of PubMed, PsycINFO, Sages Journals and Web of Science was conducted (between March and May 2021) for articles published between January 2000 and May 2021. Articles were selected after screening titles, abstracts and full texts according to our 5 inclusion criteria.

**Results:**

We included 16 articles. The diabetes, obesity and high blood pressure support programs described in these articles operate to improve physical and mental health and access to care. The approaches of these interventions are focused on the training and participation of people and the implementation of support actions adapted to the person. The majority of these interventions have a real attachment to the community.

**Conclusions:**

This review of the literature shows that support for people with chronic diseases such as diabetes, obesity or high blood pressure is based on three pillars: empowerment, peer mediation and holistic and tailor-made support for the individual. The empowerment approach, which considers the capacities and resources of individuals and whose goal is to strengthen their ability to act on their health, appears to be entirely suited to the support of these chronic diseases. This review underlines the importance of moving away from a biomedical approach to a holistic approach truly focused on the person, their capacities and their needs.

**Supplementary Information:**

The online version contains supplementary material available at 10.1186/s12889-022-14480-3.

## Background

Obesity, diabetes and high blood pressure are chronic diseases that are becoming global epidemics [1–3]. Their management is a major concern now and for the future. The treatment of these closely related diseases [4, 5] involves drug treatment but requires, above all, changes in health behaviors with regard to diet, physical activity and alcohol and tobacco consumption. The support offered to patients to engage in such changes is a public health challenge. It implies not only therapeutic patient education, focused on the control of the disease and its treatment [6, 7] but also more global support, taking into account all the facets of the person (living conditions, personality, history, etc.). These diseases affect populations differently, with a strong social gradient, being more frequent among the disadvantaged and the least educated [8, 9]. Taking into account these social inequalities is necessary while also considering the capacities and resources of individuals so that they can adopt behaviors that are favorable to their health. This is the meaning of the empowerment approach, which focuses on improving internal resources (knowledge, skills, self-confidence) and external resources (access to care) to strengthen one’s ability to act on one’s health (agency) [10–12]. The ability to grasp the information given about the illness also depends on health literacy [13]. The notion of patient support for these chronic diseases is therefore plural and can refer to therapeutic education, social support, health literacy reinforcement or empowerment.

To contribute to a better characterization of what should be comprehensive support for vulnerable populations affected by diabetes, obesity or high blood pressure, we conducted a scoping review to create an inventory and to analyze what is being proposed in terms of support for disadvantaged, immigrant or minority populations living with these chronic diseases.

## Methods

We conducted a scoping review with the aim of synthesizing the knowledge available in the literature to obtain a broad overview of the different types of support for diabetes, obesity and high blood pressure focused on vulnerable people (immigrant populations, racial or ethnic minorities or populations in precarious situations). This literature review follows the methodological recommendations of a scoping review [14–16] (Appendix 1).

### Data sources

We conducted our bibliographic search on four electronic scientific databases (PubMed, Web of Science, Sage Journals and PsychINFO). An exploratory search (snowball method based on relevant studies) allowed us to determine the most frequently used keywords in the literature and the ones closest to our subject (not all keywords are MeSH terms). The choice of these keywords was discussed between the two authors to reach an agreement. The keywords used in the four databases were as follows.

- For support: “patient education”, “self-management support”, “health promotion”
  AND: “empowerment”, “literacy”, “agency”
- For chronic diseases: “diabetes”, “high blood pressure”, “hypertension”, “obesity”
- For impact assessment: “measurement”, “assessment”, “outcomes”

The terms “self-management support,” “agency,” “high blood pressure,” “measurement,” “assessment,” and “outcomes” are not MeSH words but these keywords allowed us to narrow the search to our research topic. We decided to limit the search to the titles and abstracts of the studies to limit the scope of the search to select only those articles most relevant to our subject. The literature search was conducted between March and May 2021 by the main author. The search equations used in each database are shown in Table 1.

**Table 1:** Search equations used for the literature search

Databases	Search equation
PubMed	((“patient education”) OR (“self-management support”) OR (“health promotion”)) AND ((“empowerment”) OR (“agency”) OR (“literacy”)) AND ((“diabetes”) OR (“high blood pressure”) OR (“hypertension”) OR (“obesity”)) AND ((“assessment”) OR (“measurement”) OR (“outcomes”))
Web of Science	((AB = (patient education OR self-management support OR health promotion)) AND (AB = (empowerment OR agency OR literacy)) AND (AB = (diabetes OR high blood pressure OR hypertension OR obesity)) AND (AB = (assessment OR measurement OR outcomes))) AND DOCUMENT TYPES: (Article) Timespan: 2000–2021. Indices: SCI-EXPANDED, SSCI, A&HCI, CPCI-S, CPCI-SSH, BKCI-S, BKCI-SSH, ESCI, CCR-EXPANDED, IC.
Sage Journals	[[Abstract “patient education”] OR [Abstract “self-management support”] OR [Abstract “health promotion”]] AND [[Abstract “empowerment”] OR [Abstract “agency”] OR [Abstract “literacy”]] AND [[Abstract “diabetes”] OR [Abstract “high blood pressure”] OR [Abstract “hypertension”] OR [Abstract “obesity”]] AND [[Abstract “assessment”] OR [Abstract “measurement”] OR [Abstract “outcomes”]] Since 2000
PsychInfo	((“patient education”) OR (“self-management support”) OR (“health promotion”)) AND ((“diabetes”) OR (“high blood pressure”) OR (“hypertension”) OR (“obesity”)) AND ((“assessment”) OR (“measurement”) OR (“outcomes”)) AND ((“empowerment”) OR (“agency”) OR (“literacy”))

### Study selection

All article references were processed in Excel. Duplicates were deleted manually. Articles were then selected, first on the title and abstract and then on the full article, according to inclusion and exclusion criteria. A simple screening was performed by the main author. The inclusion criteria were determined by the two authors:

- Articles published between January 2000 and May 2021
- Articles in English
- Articles discussing interventions in the field of diabetes, obesity or high blood pressure
- Articles on an intervention targeting adults from an ethnic minority, immigrant or disadvantaged population (populations explicitly named “low incomes” or “low socioeconomic status”)
- Articles on face-to-face interventions

Articles on a pilot study were excluded when the original study was among the selected articles.

### Data extraction

The data extracted from the articles are data related to the following (Appendix 2 and 3).

- Characteristics of the articles: title, authors, date of publication, country of research
- Interventions: objectives, target population, intervention process, duration of the intervention, location of the intervention, profiles and roles of the interveners, underlying theory, main results
- Characteristics of the intervention research: study design, evaluation methodology, type of data collected, indicators, sample size

### Thematic analysis

We carried out a thematic analysis based on the data extracted from the articles, based on the following questions:

- Who is the intervention aimed at?
- What does the intervention want to do?
- How does the intervention operate?
- Who delivers the intervention and where does it take place?
- How are the objectives set evaluated?

We also analyzed the concepts underlying the interventions and their relationships with the theories of empowerment.

## Results

**Fig. 1:**
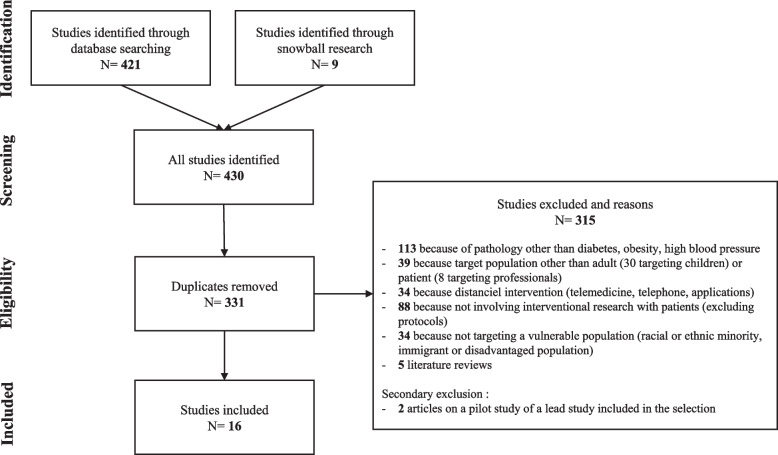
Diagram of the selection of articles included in the search

A total of 430 articles were identified in the four databases, and 16 articles that met the inclusion criteria were selected [17–32] (Fig. 1). All of the included articles were published in English; the search for French-language articles yielded no results. The interventional research described in the articles took place in the USA (11 articles), in the UK (4 articles) and in the Netherlands (1 article) (Appendix 2).

### Target audience

**Fig. 2:**
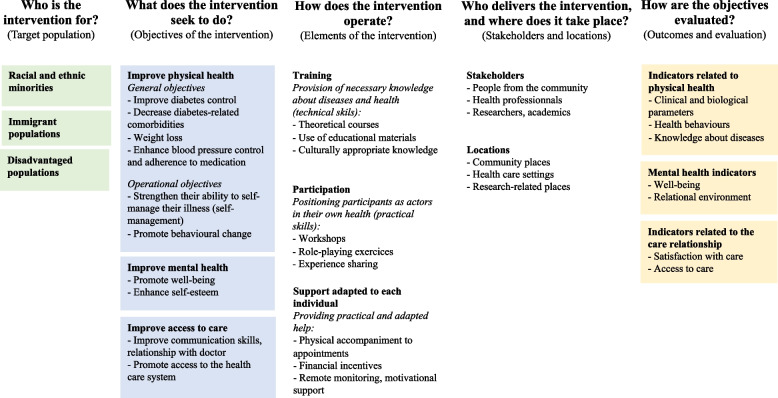
Synthesis of the intervention characteristics

The interventions described in the articles targeted people with diabetes (13 articles), obesity (2 articles) and high blood pressure (1 article) from racial or ethnic minorities in the US or The Netherlands, immigrant populations (South Asians living in the UK or US) or disadvantaged populations in the US (Fig. 2) (Appendix 4).

### Interventions aimed to improve physical and mental health and access to care

The objectives of these support programs were threefold: to improve the physical health of the participants, to improve their mental health and to improve their access to care. Improving physical health involved strengthening participants’ ability to manage their illness and health (self-management) (e.g., being independent in taking medication, self-monitoring of blood sugar) and involved behavioral change (toward adopting health-promoting behaviors, such as adopting a healthy diet, engaging in regular physical activity).

More generally, interventions focused on diabetes aimed to improve control of the disease and reduce associated comorbidities, while interventions focused on obesity aimed at weight loss. Improving mental health involved actions promoting well-being and strengthening self-esteem (around the issues of “living well with the disease,” the use of self-praise to maintain health-promoting behaviors, and the management of emotions, stress and depression). Finally, improving access to care involved improving communication skills with caregivers (promoting shared decision-making) and improving access to the care system (support in the care system, strengthening the link between the community and health professionals).

### Training, participation and case-by-case support as principles of action

The support programs relied upon three categories of actions (Fig. 2).

- The training of participants to give them technical skills: theoretical courses to deliver knowledge about diseases and health in general, with the use of written or audiovisual educational material.
- Active participation to acquire practical skills and experiential knowledge: participants are positioned as actors in their own health through workshops to learn how to cook, shop or exercise, through group exercises or role plays to work on problem solving, emotional management, sharing experiences and setting individual goals to achieve.
- Support tailored to each individual case: providing practical help adapted to the needs of each participant, such as physical accompaniment to appointments, financial incentives (e.g., a voucher to buy from a market gardener), interventions adapted to the literacy level, follow-up and motivational support after the intervention.

### Community-based interventions

The facilitators who deliver the intervention are mostly people from the community targeted by the intervention (Appendix 4), and they share the same language and culture. They are community members or leaders trained in therapeutic patient education for diabetes or obesity or peer educators (i.e., people themselves affected by the disease) trained in therapeutic education for these conditions or health professionals from the community. In other interventions, health professionals (diabetes nurses, dieticians, chiropodists, doctors) or, more rarely, researchers or academics, carry out the actions. Interventions are most often carried out in places linked to the community (community centers, participants’ homes) or in health centers (doctors’ surgeries, health centers or clinics).

### An evaluation that relies primarily on biomedical indicators

The indicators used to evaluate the impact of the interventions are of various kinds. Reflecting the objectives of the interventions, we identified three types of indicators (Fig. 2): indicators related to physical health, indicators related to mental health and indicators related to the care relationship.

The physical health indicators are:

- Clinical and biological parameters (biological markers: HbA1c, blood glucose monitoring, HDL, LDL, blood lipids; blood pressure; weight/BMI, waist circumference; diabetes complications).
- Behavioral indicators (health and care behaviors related to diabetes management; preventive health behaviors; problem solving; self-efficacy; motivation and barriers; treatment adherence; diabetes empowerment level).
- Disease knowledge indicators.

Mental health-related indicators are as follows:

- Indicators of well-being and social relationships (quality of life; social support; mental health).

Indicators related to the health care relationship are:

- Indicators of satisfaction, access to care.

All interventions were evaluated on clinical and biological indicators (Appendix 4). They have the advantage of being objective indicators that are simple to measure and comparable. Some of them require a time lag of several months before they can be observed. Indicators relating to behavior and self-esteem are less used and are subjective indicators (declarative).

### The conceptual reference to empowerment

Half of the articles make explicit reference to empowerment theories: the “Health Self-Empowerment Theory” (HSET) [18, 21] developed by Tucker et al. [33] and an “Anderson and Funnell concept of empowerment” [19, 20, 23, 31, 34]. The objectives developed in these theories are to strengthen the knowledge of individuals (theoretical knowledge, experiential knowledge) which enables them to make informed decisions and/or decisions based on experience (one’s own or that of others); to strengthen the problem-solving abilities of individuals (how to put into practice self-management techniques for one’s illness) in order to adopt or maintain behaviors favorable to one’s health; strengthening or maintaining “internal” motivation (i.e., motivation that comes from within the person and not from outside) to change one’s behavior and adopt health-promoting behaviors and encouraging the identification and management of one’s emotions when faced with difficult situations.

## Discussion

This work contributes to the literature on diabetes, obesity and high blood pressure support methods. This scoping review shows us that 1) the interventions aim to strengthen the personal resources (both technical and experiential) of individuals to understand and manage their disease, 2) they are rooted in the community of the people concerned, and 3) they involve both comprehensive (taking into account all the dimensions of the person) and individualised (tailor-made) support.

The main result of this review is that the selected interventions have common objectives of improving physical and mental health and access to care and modes of action that are based on the training and participation of participants and tailored support, all of which are part of the notion of empowerment in health.

Half of the articles made explicit reference to empowerment theories, and in the majority of articles, even if no empowerment theory was cited, we found elements of the empowerment process as theorised by Kabeer, Ninacs and Karp [10–12]. Indeed, the empowerment process can only take place if the individual has the possibility to make a choice, i.e., if the individual has the necessary resources at their disposal so that they can make a choice. The interventions we have studied meet this empowerment requirement by providing the necessary internal and external resources to the participants. The internal resources are the reinforcement of theoretical knowledge about diseases and health and the reinforcement of practical skills (self-management of treatment, balanced cooking, physical exercise), and the external resources are the provision of support, for example, through vouchers, accompaniment to medical appointments and thus access to the health care system. Once the resources are available to the person and therefore the possibility exists for them to make a choice, the empowerment process is then possible, and it is favored by the place that the individual will take in the collective. In the interventions, it is through participation in group workshops and by speaking out and sharing experiences that individuals will take their place within a group and strengthen their self-confidence and self-esteem. All these keys (resources and participation) will enable the individual to exercise choice, to make decisions for their own health in light of their knowledge, and finally to act on their decision [10]. In the present case of support for people affected by diabetes or obesity, the interventions aim to produce this empowerment process to strengthen their ability to manage their disease and their health, to improve diabetes and high blood pressure control and obesity management, and to reduce comorbidities.

The second point is the embedding of interventions in the community targeted by the intervention. Many interventions involve people from the community to which the intervention is directed in leading the actions. Even if it is not explicitly named, health mediation, in the sense of an interface function between vulnerable people who are far from the health care system and the professionals involved in their health pathway to support a process of autonomy in health [35, 36], is widely present in these interventions. The 2019 WHO report on the roles and effectiveness of intercultural mediators in health care in the context of access to care for migrant populations in Europe and North America [37] highlights six areas of expertise of the mediator role: “providing interpretation; bridging socio-cultural gaps; preventing conflict and promoting conflict resolution; supporting integration into health systems, supporting empowerment and advocacy; building trust and facilitating the therapeutic relationship; providing psychosocial support, education and health promotion.” In the interventions studied, we found elements of this concept of health mediation, both in the profile of the facilitators (people from the community targeted by the intervention, health professionals, community members or peers trained in therapeutic education); their roles (leading diabetes education sessions, accompanying participants to medical appointments, facilitating communication between caregivers and patients); the way in which the actions are organized (locations and times adapted to the lifestyle of the target groups); and finally the adaptation of the interventions to the culture and language. For these population groups, which generally have a poorer quality of care than the majority population of the country or with a favorable socioeconomic standard of living, health mediation makes it possible to overcome financial and administrative barriers, linguistic barriers and cultural barriers (lack of knowledge on the part of both patients and health professionals) to access quality care. Health mediation seems to be a means of fostering empowerment at the individual level in the sense that it allows better access to resources (access to quality health and preventive care, adapted to their level of understanding, lifestyle, culture, etc.). In France, the *Haute Autorité de Santé* defines one of the objectives of mediation in health as being the reinforcement of “the autonomy and capacity to act of people in the management of their health” [38]. Mediation can also be a means of promoting collective empowerment. In some studies [18, 21, 29, 30], community members are also integrated into the research process from the outset (in community-based participatory research). The active participation of community members in the research process promotes a process of community empowerment [39].

Finally, the third point derived from this review is the fact that in several interventions [18, 20, 22–24, 30, 31], the support proposed is individualized and comprehensive. The support is adapted to each individual case and takes into account the different facets of the person’s life: their physical and mental health, their economic and social situation and their social support. Indeed, the support provided to participants through financial assistance (financial incentives, vouchers, etc.), physical accompaniment to medical appointments, telephone follow-up (motivational support to maintain efforts, reminders of appointments), and home visits are designed through individual interviews with the workers to identify specific barriers to behavioral change and to set objectives to be reached, chosen by the participant. These actions are the markers of a global and individualised accompaniment of the person, which corresponds to a holistic patient-centered approach. The patient-centered care approach is defined by the *Institute of Medicine* as “care that respects and responds to each patient’s preferences, needs, and values, and ensures that the patient’s values guide all clinical decisions” [40]. This approach has been shown to be relevant for the best support of people affected by chronic disease [41]. In the case of obesity, for example, even before pursuing treatment goals, health care providers must consider the patient holistically to understand their needs, the changes they are motivated to make, and to identify the barriers that might hinder weight loss, the improvement of their comorbidities and quality of life. The French *Haute Autorité de Santé* defines the first stage of therapeutic patient education (TPE) as follows: “get to know the patient, identify their needs, expectations and receptiveness to the TPE proposal; apprehend the different aspects of the patient’s life and personality, evaluate their potential, take into account their requests and project; apprehend the patient’s way of reacting to their situation and their personal, social and environmental resources” [42].

A limitation of our review is that it did not select many studies of programs specifically dedicated to supporting high blood pressure and obesity (1 and 2 articles out of 16 in total, respectively). These results lead us to suggest that high blood pressure and obesity are less frequently identified as chronic pathologies requiring global therapeutic support, such as proposed for diabetes. Although the European Commission has recognized obesity as a chronic disease, this is not the case in all European countries, and its implementation in health systems raises many financial and operational issues [43]. For example, in France, only diabetes is recognized by the Health Insurance as a “Long-Term Condition.” This status, in addition to the total coverage of medical costs by social security, is a form of recognition by society of these chronic pathologies.

Another limitation concerns the scope of our research. We know that there are other articles in the literature examining the support of people with diabetes, hypertension or obesity, but if the concepts of empowerment, agency or literacy are not mentioned in the title or abstract, these articles were not selected. Interventions aimed at strengthening self-management implicitly reinforce people’s empowerment, but this would need to be explicitly described. This limitation explains the small number of articles selected and may also explain why support programs taking place mostly in the US and the UK have been selected, even though we are aware that support programs are taking place in other countries. Moreover, the gray literature was not taken into account in this bibliographical search, which may exclude a number of interventions carried out and evaluated by field workers that have not been studied and published in international scientific journals.

Finally, we point out that this research was carried out by the authors without the help of an academic librarian. We carried out an exploratory study (snowball method from relevant studies) to determine the keywords closest to our theme (all the keywords are not MeSH terms), and screening and extraction process was performed by one reviewer only.

## Conclusion

In conclusion, this review of the literature shows that support for people with chronic diseases such as diabetes, obesity or high blood pressure is based on three pillars: empowerment, peer mediation and holistic and tailored support for the person. Although not explicitly named, empowerment is omnipresent both as a process and as an objective to be achieved in support programs through training, participation and support adapted to the person. This empowerment approach for people living with chronic disease is based on peer mediation in health (sharing the same culture or language or sharing the same disease), which makes sense in the present case of support for disadvantaged populations. Some programs go even further by integrating these mediators into the research process (participatory research), thus completing the empowerment process at a collective and community level. Finally, this work shows us that support for chronic disease cannot be provided without taking into account the person as a whole and in their uniqueness. This review underlines the importance of moving away from a biomedical approach that goes from the doctor to the patient to a holistic approach that is truly centered on the person, their capacities and needs, since health is a state of general well-being and not just the absence of disease. The current health crisis period linked to the COVID-19 pandemic, where biomedical approaches have often taken precedence over the consideration of social and mental health aspects, reminds us of this need.

To further develop this scoping review and help practitioners develop effective programs, it would be relevant to deepen this work by a literature review focusing on the effectiveness of these programs. More broadly, it would be relevant to develop research to evaluate the impact of such programs on the health of individuals, on their satisfaction and on their ability to take charge of their health.

## Supplementary Information

**Additional file 1.**

**Additional file 2.**

**Additional file 3.**

**Additional file 4.**

## Data Availability

All data generated or analyzed during this study are included in this published article (Appendix 2 and 3).

## References

[1] Saeedi P, Petersohn I, Salpea P, Malanda B, Karuranga S, Unwin N (2019). Global and regional diabetes prevalence estimates for 2019 and projections for 2030 and 2045: Results from the International Diabetes Federation Diabetes Atlas, 9th edition. Diabetes Res Clin Pract.

[2] Obesity [Internet]. WHO [cited 2021 Dec 23]. Available from: https://www.who.int/health-topics/obesity#tab=tab_1.

[3] Hypertension [Internet]. WHO [cited 2021 Dec 23]. Available from: https://www.who.int/health-topics/hypertension#tab=tab_1.

[4] has-santé.fr [Internet]. Haute Autorité de Santé [cited 2021 Nov 30]. Available from: https://www.has-sante.fr/upload/docs/application/pdf/2015-02/7v_referentiel_2clics_diabete_060215.pdf.

[5] Prise en charge de l’hypertension artérielle de l’adulte [Internet]. Haute Autorité de Santé. [Management of high blood pressure in adults [Internet]. High Authority on Health.] [cited 2021 Nov 30]. Available from: https://www.has-sante.fr/jcms/c_2059286/fr/prise-en-charge-de-l-hypertension-arterielle-de-l-adulte.

[6] WHO (1998). Therapeutic patient education: continuing education programmes for health care providers in the field of prevention of chronic diseases: report of a WHO working group.

[7] Newman S, Steed L, Mulligan K (2004). Self-management interventions for chronic illness. Lancet.

[8] Santé Publique France. Le poids du diabète en France en 2016. Synthèse épidémiologique. 2018;8 [Santé Publique France. The burden of diabetes in France in 2016. Epidemiological synthesis. 2018;8.].

[9] Santé Publique France. Étude de santé sur l’environnement, la biosurveillance, l’activité physique et la nutrition (Esteban), 2014–2016. Volet Nutrition. Chapitre Corpulence. :43. [Internet]. [Santé Publique France. Environment, biomonitoring, physical activity and nutrition health study (Esteban), 2014–2016. Nutrition component. Corpulence chapter. :43.] [Internet]. [cited 2022 Nov 7]. Available from: https://www.santepubliquefrance.fr/determinants-de-sante/nutrition-et-activite-physique/etude-de-sante-sur-l-environnement-la-biosurveillance-l-activite-physique-et-la-nutrition-esteban-2014-2016.-volet-nutrition.-chapitre-corpulence.

[10] Ninacs WA (2005). Empowerment et service social : approches et enjeux. Serv Soc.

[11] Kabeer N (1999). Resources, agency, achievements: reflections on the measurement of Women’s empowerment. Dev Chang.

[12] Karp C, Wood SN, Galadanci H, Sebina Kibira SP, Makumbi F, Omoluabi E (2020). ‘I am the master key that opens and locks’: presentation and application of a conceptual framework for women’s and girls’ empowerment in reproductive health. Soc Sci Med.

[13] Nutbeam D (2000). Health literacy as a public health goal: a challenge for contemporary health education and communication strategies into the 21st century. Health Promot Int.

[14] Arksey H, O’Malley L (2005). Scoping studies: towards a methodological framework. Int J Soc Res Methodol.

[15] Levac D, Colquhoun H, O’Brien KK (2010). Scoping studies: advancing the methodology. Implement Sci.

[16] Tricco AC, Lillie E, Zarin W, O’Brien KK, Colquhoun H, Levac D (2018). PRISMA extension for scoping reviews (PRISMA-ScR): checklist and explanation. Ann Intern Med.

[17] Hill-Briggs F, Lazo M, Peyrot M, Doswell A, Chang YT, Hill MN (2011). Effect of Problem-Solving-Based Diabetes Self-Management Training on Diabetes Control in a Low Income Patient Sample. J Gen Intern Med..

[18] Tucker CM, Lopez MT, Campbell K, Marsiske M, Daly K, Nghiem K (2014). The effects of a culturally sensitive, empowerment-focused, community-based health promotion program on health outcomes of adults with type 2 diabetes. J Health Care Poor Underserved.

[19] Tang T, Funnell M, Oh M. Lasting Effects of a 2-Year Diabetes Self-Management Support Intervention: Outcomes at 1-Year Follow-Up. Prev Chronic Dis. 2012; [cited 2021 Sept 8]; Available from: http://www.cdc.gov/pcd/issues/2012/11_0313.htm. 10.5888/pcd9.110313 PMC3457752 22677159

[20] Peek ME, Harmon SA, Scott SJ, Eder M, Roberson TS, Tang H (2012). Culturally tailoring patient education and communication skills training to empower African-Americans with diabetes. Transl. Behav Med.

[21] Tucker CM, Wippold GM, Williams JL, Arthur TM, Desmond FF, Robinson KC (2017). A CBPR study to test the impact of a church-based health empowerment program on health behaviors and health outcomes of black adult churchgoers. J Racial Ethn Health Disparities.

[22] Fernandes R, Chinn CC, Li D, Halliday T, Frankland TB, Wang CMB, Wang Z, Morioka M, Arndt RG, Ozaki RR (2018). Financial Incentives for Medicaid Beneficiaries With Diabetes: Lessons Learned From HI-PRAISE, an Observational Study and Randomized Controlled Trial. Am J Health Promot..

[23] Anderson RM, Funnell MM, Nwankwo R, Gillard ML, Oh M, Fitzgerald JT (2005). Evaluating a problem-based empowerment program for African Americans with diabetes: results of a randomized controlled trial. Ethn Dis.

[24] Sorkin DH, Mavandadi S, Rook KS, Biegler KA, Kilgore D, Dow E (2014). Dyadic collaboration in shared health behavior change: the effects of a randomized trial to test a lifestyle intervention for high-risk Latinas. Health Psychol.

[25] Hawthorne K (2001). Effect of culturally appropriate health education on glycaemic control and knowledge of diabetes in British Pakistani women with type 2 diabetes mellitus. Health Educ Res.

[26] Vyas A, Haidery AZ, Wiles PG, Gill S, Roberts C, Cruickshank JK (2003). improve knowledge, awareness and self- management among South Asians with diabetes in Manchester.

[27] Bellary S, O’Hare J, Raymond N, Gumber A, Mughal S, Szczepura A (2008). Enhanced diabetes care to patients of south Asian ethnic origin (the United Kingdom Asian diabetes study): a cluster randomised controlled trial. Lancet.

[28] Choudhury SM, Brophy S, Fareedi MA, Zaman B, Ahmed P, Williams R (2009). Examining the effectiveness of a peer-led education programme for type 2 diabetes and cardiovascular disease in a Bangladeshi population. Diabet Med.

[29] Islam NS, Wyatt LC, Patel SD, Shapiro E, Tandon SD, Mukherji BR (2013). Evaluation of a community health worker pilot intervention to improve diabetes Management in Bangladeshi Immigrants with Type 2 diabetes in new York City. Diabetes Educ.

[30] Trevisi L, Orav JE, Atwood S, Brown C, Curley C, King C (2019). Integrating community health representatives with health care systems: clinical outcomes among individuals with diabetes in Navajo nation. Int J Equity Health.

[31] Spencer MS, Kieffer EC, Sinco B, Piatt G, Palmisano G, Hawkins J (2018). Outcomes at 18 months from a community health worker and peer leader diabetes self-management program for Latino adults. Diabetes Care.

[32] Beune EJAJ, Moll van Charante EP, Beem L, Mohrs J, Agyemang CO, Ogedegbe G (2014). Culturally adapted hypertension education (CAHE) to improve blood pressure control and treatment adherence in patients of African origin with uncontrolled hypertension: cluster-randomized trial. PLoS One.

[33] Tucker CM, Roncoroni J, Wippold GM, Marsiske M, Flenar DJ, Hultgren K (2018). Health self-empowerment theory: predicting health behaviors and BMI in culturally diverse adults. Fam Community Health.

[34] Funnell MM, Anderson RM (2004). Empowerment and self-Management of Diabetes. Clin Diabetes.

[35] Haschar-Noé N, Bérault F (2019). La médiation en santé : une innovation sociale ? Obstacles, formations et besoins. Santé Publique.

[36] Décret n° 2017–816 du 5 mai 2017 relatif à la médiation sanitaire et à l’interprétariat linguistique dans le domaine de la santé. 2017–816 May 5, 2017. [Decree No. 2017–816 of May 5, 2017, relating to health mediation and language interpreting in the health field. 2017–816 May 5, 2017.] Available from: https://www.legifrance.gouv.fr/jorf/id/JORFTEXT000034602662/.

[37] Hanswhorofe V (2019). What are the roles of intercultural mediators in health care and what is the evidence on their ... Contributions and effectiveness in improving acces. Place of publication not identified.

[38] Haute Autorité de santé. La médiation en santé pour les personnes éloignées des systèmes de prévention et de. 2017; soins 70. [High Health Authority. Health mediation for people far from the prevention and care systems. 2017; care 70.]. [cited 2022 Nov 7]. Available from: https://www.has-sante.fr/jcms/c_2801497/fr/la-mediation-en-sante-pour-les-personnes-eloignees-des-systemes-de-prevention-et-de-soins.

[39] Ninacs WA. Empowerment: cadre conceptuel et outil d’évaluation de l’intervention sociale et communautaire. Québec (Canada), La Clé. 2003;28. [cited 2022 Nov 7]. Available from: http://envision.ca/pdf/w2w/Papers/NinacsPaper.pdf.

[40] Institute of Medicine (2001). Crossing the quality chasm: a new health system for the 21st century.

[41] Fastenau J, Kolotkin RL, Fujioka K, Alba M, Canovatchel W, Traina S (2019). A call to action to inform patient-centred approaches to obesity management: development of a disease-illness model. Clin Obes.

[42] Haute Autorité de Santé. Éducation thérapeutique du patient Définition, finalités et organisation: 2007 Jun. Obésité. 2009 Mar;4(1):39–43. [High Health Authority. Therapeutic patient education Definition, aims and organization: 2007 Jun. Obesity. 2009 Mar;4(1):39–43.]. [cited 2022 Nov 7]. Available from: https://www.has-sante.fr/upload/docs/application/pdf/etp_-_definition_finalites_-_recommandations_juin_2007.pdf.

[43] Burki T (2021). European Commission classifies obesity as a chronic disease. Lancet Diabetes Endocrinol.

