# Supplementary material for: Empowerment-based support program for vulnerable populations living with diabetes, obesity or high blood pressure: a scoping review
Source: BMC Public Health. 2022 Nov 9;22:2051. doi: 10.1186/s12889-022-14480-3 (PMC9644395; doi:10.1186/s12889-022-14480-3)
Supplement: Supplementary file 1 — Additional file 1. [file 12889_2022_14480_MOESM1_ESM.docx]

**Appendix 1: Preferred Reporting Items for Systematic reviews and Meta-Analyses extension for Scoping Reviews (PRISMA-ScR) Checklist**

| **SECTION** | **ITEM** | **PRISMA-ScR CHECKLIST ITEM** | **REPORTED ON PAGE #** |
| --- | --- | --- | --- |
| **TITLE** | | | |
| Title | 1 | Empowerment-based support program for vulnerable populations living with diabetes, obesity or high blood pressure: a scoping review. | 1 |
| **ABSTRACT** | | | |
| Structured summary | 2 | **Background:** To provide an inventory and an analysis of the different types of support for chronic diseases such as diabetes, high blood pressure and obesity among disadvantaged, immigrant or minority populations in order to contribute to a better definition and characterization of what should be a global support for these vulnerable populations for these diseases.  **Methods:** Relevant literature published between January 2000 and May 2021 has been reviewed. Articles were selected after screening titles, abstracts and full texts according to our five inclusion criteria.  **Results:** Of the 430 titles identified, 16 articles were included in this scoping review. Interventions described in these articles aim to improve physical and mental health and access to care by approaches focused on training and participation of people and the implementation of support actions adapted to the person.  **Conclusion:** Support for people with chronic diseases such as diabetes, high blood pressure or obesity is based on three pillars: empowerment, peer mediation and holistic and tailor-made support for the individual. The empowerment approach appears to be entirely suited to the support of these chronic diseases. | 1-2 |
| **INTRODUCTION** | | | |
| Rationale | 3 | The management of chronic diseases such as diabetes, obesity and high blood pressure is a major global challenge, particularly among the most disadvantaged populations. Beyond the biomedical management of these diseases, comprehensive support that takes into account the economic and social situation of people is fundamental. The empowerment approach, which considers the capacities and resources of individuals and whose aim is to strengthen their ability to act on their health, seems to be perfectly suited to the support of these chronic diseases. | 3 |
| Objectives | 4 | The aim of the scoping review is to make an inventory and analyze what is on offer in terms of support for people affected by diabetes, obesity or high blood pressure in disadvantaged, immigrant or minority populations, in order to contribute to a better definition and characterization of what should be comprehensive support for these vulnerable populations for these diseases. | 3 |
| **METHODS** | | | |
| Protocol and registration | 5 | No protocol available. |  |
| Eligibility criteria | 6 | Inclusion period:  - Dates: between January 2000 and May 2021  Language:  - English  Field of interventions:  - Interventions in the field of diabetes, obesity or high blood pressure management  Target population:  - Vulnerable populations : ethnic minorities, immigrant populations, disadvantaged population  Type of support:  - Face-to-face intervention | 5 |
| Information sources | 7 | Databases: PubMed, Web of Science, PsychINFO, Sage Journals | 4 |
| Search | 8 | Keywords: patient education, self-management support, health promotion, diabetes, high blood pressure, hypertension, obesity, assessment, measurement, outcomes, empowerment, agency, literacy  Pub Med search equation: (("patient education") OR ("self-management support") OR (“health promotion”)) AND (("diabetes") OR ("high blood pressure") OR ("hypertension") OR ("obesity")) AND ((“assessment”) OR (“measurement”) OR (“outcomes”)) AND ((“empowerment”) OR (“agency”) OR (“literacy”))  Limits: January 2000 to May 2021, title and abstract | 5 |
| Selection of sources of evidence | 9 | The identified articles were processed by Excel by the main author, duplicates were deleted manually. Articles were selected on the basis of inclusion and exclusion criteria, first on titles and abstracts, then on full articles. | 5 |
| Data charting process | 10 | Data entry was done on an Excel table by the main author. | 5 |
| Data items | 11 | Characteristics of the articles: title, authors, date of publication, country of research  Interventions: objectives, target population, intervention process, duration of the intervention, location of the intervention, profiles and roles of the interveners, underlying theory, main results  Characteristics of the intervention research: study design, evaluation methodology, type of data collected, indicators, sample size | 6 |
| Critical appraisal of individual sources of evidences | 12 | Not realised. |  |
| Synthesis of results | 13 | We carried out a thematic analysis based on the data extracted from the articles, based on the following questions:  - Who is the intervention for?  - What does the intervention want to do?  - How does the intervention operate?  - Who delivers the intervention and where does it take place?  - How are the objectives set evaluated? | 6 |
| **RESULTS** | | | |
| Selection of sources of evidence | 14 | Of the 430 articles identified, 16 were selected (Figure 1). | 6-7 |
| Characteristics of sources of evidence | 15 | The characteristics of the articles are summarized in Appendix 2 and Appendix 3. The interventions described in the articles targeted people with diabetes (13 articles), obesity (2 articles) and high blood pressure (1 article) from racial or ethnic minorities in the US or The Netherlands, immigrant populations (South Asians living in the UK or US) or disadvantaged populations in the US). | 7 |
| Critical appraisal within sources of evidence | 16 | Not realised. |  |
| Results of individual sources of evidence | 17 | The diabetes, high blood pressure and obesity support programs described in these articles aim to improve physical and mental health and access to care. The modes of action of these interventions are focused on training and participation of people and the implementation of support actions adapted to the person. Majority of these interventions have a real attachment in the community | 7-11 |
| Synthesis of results | 18 | This scoping review shows us that 1) the interventions aim to strengthen the personal resources of individuals to understand and manage their illness, 2) they are rooted in the community of the people concerned, and 3) they involve both comprehensive (taking into account all the dimensions of the person) and individualized (tailored) support. | 7-11 |
| **DISCUSSION** | | | |
| Summary of evidence | 19 | This review of the literature demonstrates that support for people with chronic diseases such as diabetes or obesity is based on three pillars: empowerment, peer mediation and holistic and tailored support for the individual. Although not explicitly named, empowerment is omnipresent both as a process and as a goal to be achieved in the support programs through training, participation and support tailored to the individual. | 11-14 |
| Limitations | 20 | A limitation of our review is that it did not select many studies of programs specifically dedicated to supporting high blood pressure and obesity (1 and 2 articles out of 16 in total, respectively). These results lead us to suggest that high blood pressure and obesity are less frequently identified as chronic pathologies requiring global therapeutic support, such as proposed for diabetes.  Another limitation concerns the scope of our research. We know that there are other articles in the literature examining the support of people with diabetes, hypertension or obesity, but if the concepts of empowerment, agency or literacy are not mentioned in the title or abstract, these articles were not selected.  Finally, we point out that this research was carried out by the authors without the help of an academic librarian. We carried out an exploratory study (snowball method from relevant studies) to determine the keywords closest to our theme, and screening and extraction process was performed by one reviewer only. | 14-15 |
| Conclusions | 21 | This review underlines the importance of moving away from a biomedical approach from the doctor to the patient towards a holistic approach that is truly centered on the person and their needs, since health is a state of general well-being and not just the absence of disease. The current period of health crisis linked to the Covid-19 pandemic, where biomedical approaches have taken precedence over the consideration of social and mental health aspects, confirms this need.  To further develop this scoping review and help practitioners develop effective programs, it would be relevant to deepen this work by a literature review focusing on the effectiveness of these programs. | 15-16 |
| **FUNDING** | | | |
| Funding | 22 | This research is part of Julia Eïd's PhD in public health, financed by a Cifre convention (*Convention Industrielle de Formation par la Recherche*), by the Ikambere association and by the Nehs foundation. | 17 |
