# Supplementary material for: Empowerment-based support program for vulnerable populations living with diabetes, obesity or high blood pressure: a scoping review
Source: BMC Public Health. 2022 Nov 9;22:2051. doi: 10.1186/s12889-022-14480-3 (PMC9644395; doi:10.1186/s12889-022-14480-3)
Supplement: Supplementary file 2 — Additional file 2. [file 12889_2022_14480_MOESM2_ESM.docx]

**Appendix 2: Characteristics of included articles and interventions**

| **Reference** | **Author and year of publication** | **Country of research** | **Targeted disease** | **Intervention objectives** | **Intervention process** | **Intervention duration** | **Intervention location** | **Stakeholder profile and role** | **Intervention theory** |
| --- | --- | --- | --- | --- | --- | --- | --- | --- | --- |
| 17 | Hill et al, 2011 | USA | Diabetes | Train participants in problem solving to promote diabetes self-management, clinical monitoring parameters and health behaviors, in an African American and low-income population in the United States. | Intensive program: one diabetes and cardiovascular diseases (CDV) education session and eight problem-solving training sessions adapted for low literacy. The module targeted awareness of CVD risk factors; knowledge of clinical targets for A1C, blood pressure, and cholesterol; and self-management behaviors of taking medication, self-monitoring, healthy eating, and getting regular physical activity.  Condensed program: one Diabetes and DVD education session and one problem-solving training session | One month versus one week | *Not specified* | *Not specified* | The D’Zurilla and Nezu problem-solving therapy |
| 18 | Tucker et al, 2014 | USA | Diabetes | Improving the health and well-being of people with diabetes through a series of three health promotion workshops in a low-income population in the United States | Workshop 1 of the health promotion program included (a) didactic presentations to teach participants health-promoting behaviors and how to use self-help to maintain these behaviors, (b) demonstrations by a nutritionist on how to read and understand nutrition labels, (c) demonstrations on how to shop for and prepare culturally-related desired meals in a healthier way without sacrificing taste, (d) breakout sessions with psychologists and graduate students in psychology, in which research team members and participants shared practical and culturally appropriate strategies for adopting health-promoting behaviors and overcoming barriers to adopting such behaviors, as well as strategies for reducing stress and depression-emotions that often contribute to and result in unhealthy eating and inactivity.  Workshop 2 of the health promotion program focused on training participants to use cognitive behavioral skills and specific behaviors and strategies to elicit desired health care behaviors from their providers and other health care staff. | 2 weekly sessions, then 3 weeks follow-up | At a large university and at a local health care clinic | A large, ethnically diverse research team:  research faculty members, community member consultants, community health care providers, graduate research assistants, and undergraduate research assistants | Health Self-Empowerment Theory |
| 19 | Tang et al, 2012 | USA | Diabetes | Improving clinical parameters, self-care and psychosocial health status through a 2-year diabetes self-management intervention, in an African American population in the United States. | Groups (88 weekly sessions) led by 2 health care professionals (nurse and psychologist) and emphasized experiential learning, emotional coping, problem solving, goal setting, and action planning; group discussion was guided by participant- identified self-management priorities and concerns  -Phase 1: a 6-month mailed DSME intervention with clinical feedback  -Phase 2: a 2-year ongoing DSMS intervention called the Lifelong Diabetes Self-Management Intervention  - Phase 3: a post-DSMS intervention follow-up period in which participants received usual care and no intervention | 24 months | At a local community center | A nurse certified diabetes educator and a clinical psychologist | Anderson and Funnell’s empowerment conception |
| 20 | Peek et al, 2012 | USA | Diabetes | To improve diabetes self-efficacy, self-care behaviors and clinicals parameters of participants with diabetes through a culturally appropriate diabetes education and shared decision training intervention, in an African American population in the United States. | The first six sessions consisted of general diabetes education, the next three sessions focused on shared decision-making and patient/provider communication, and the last session provided a review of curricular materials and additional opportunities to practice self-care and SDM skills  The team created a mock grocery store based on the labels and packaging of food items available in locally utilized grocery stores which were donated by class participants | 10 weeks, then quarterly follow-up for 6 months | In a federally qualified health center and in a mock grocery store | A nurse certified diabetes educator, a registered dietician and a primary care physician diabetes. And a diabetes support group with patient-led and peer support | “Patient empower model” |
| 21 | Tucker et al, 2016 | USA | Obesity | Increase the level of physical activity and healthy eating, and decrease the BMI of overweight and obese people through a church-based program to increase health-smart behaviors, in a black American population in New York. | A goal-setting session (individualized coaching) during week 1, followed by four weekly, 90-min meeting sessions that were led by two of the health empowerment coaches, and the final week consisted of a provider and community member health panel. | 6 weeks | In four churches | The pastors, the four church leaders (they were trained as health empowerment coaches), community members and a panel of professionals (physicians, dieticians, nutritionists, psychologists and physical fitness experts) | Health Self-Empowerment Theory |
| 22 | Fernandes et al, 2017 | USA | Diabetes | Improving health behaviors and health status of people with diabetes through a financial incentive-based intervention in Hawaii. | Participants included in the study received financial incentives; they could earn a maximum of $320 per year. Each Federally qualified health center determined the optimal type of financial incentives to motivate and meet the needs of their patients. Preferred options were gift cards to grocery stores, pharmacies, gas stations, or retail stores. Less common forms of incentives were vouchers for farmers' markets, massages, or food. | 1 year at least | Federally qualified health centers | Clinicians and community health workers | *Not specified* |
| 23 | Anderson et al, 2005 | USA | Diabetes | Strengthening diabetes self-management, improving clinical outcomes through an empowerment-based intervention for African Americans with type 2 diabetes. | The intervention consisted of six weekly two-hour group sessions: reflecting on self-management experiments; discussing the emotional experience of living with diabetes; engaging in systematic problem-solving; answering clinical questions; culturally tailored education materials. After their six weeks of sessions all patients were offered the opportunity to join a monthly support group or receive a monthly phone call. | 6 weeks, then a monthly follow-up for one year | In convenient community-based locations | A nurse and a dietician | An “empowerment approach” |
| 24 | Sorkin et al, 2014 | USA | Obesity | Promoting weight loss and improving dietary behaviors of women who are obese or at high risk of developing obesity through a behavioral lifestyle intervention targeting the mother-daughter dyad in a Mexican American population. | The 16-week Unidas intervention consisted of the following: four group meetings, eight home visits, and booster telephone calls by a lifestyle community coach. | 16 weeks | Federally qualified health centers and home visits | A lifestyle community coach | *Not specified* |
| 25 | Hawthorne, 2001 | UK | Diabetes | Improving diabetes knowledge and outcomes through a diabetes education intervention in a Pakistani population living in the UK. | The intervention consists on one-to-one structured diabetes health education, delivered by a link worker with pictorial flash-cards as a visual aid; and focus group discussions about an appropriate diabetic diet, the value of glucose monitoring (and how to act on the results), diabetic complications (and how to limit their onset and progression), and services offered by the diabetic clinic. | Half a day | In a hospital clinic, at practitioners’ surgeries and home visits | A link worker | *Not specified* |
| 26 | Vyas et al, 2003 | UK | Diabetes | To improve diabetes knowledge, awareness and self-management through a diabetes education intervention in GP practices in a South Asian population living in the UK. | Patients in intervention practices were invited to special clinic sessions on four occasions over the year, were seen at least once by each of the following: diabetes specialist nurse, podiatrist and dietician, and received an individual diabetes education. | 1 year | In medical offices and clinics where patients do their diabetes follow-up | Diabetes specialist nurse, podiatrist and dietician, health professional who speak the language or interpreters | *Not specified* |
| 27 | Bellary et al, 2008 | UK | Diabetes | Improving cardiovascular risk factors in people with diabetes using a culturally sensitive intervention to improve the care package in general practice cabinets, in a South Asian population living in the UK. | The intervention consisted of an additional practice nurse time (4h per practice per week), supported by link workers and a community nurse specializing in diabetes. | 2 years | In medical offices where patients do their diabetes follow-up | Link workers and community nurse specializing in diabetes | *Not specified* |
| 28 | Choudhury et al, 2008 | UK | Diabetes | Improving diabetes knowledge and health behaviors of people with diabetes through a diabetes education intervention in a Bangladeshi population living in the UK. | The educational session in this study was done over one 4-h session, with a shopping tour (was to give participants an overview of the different types of foods and their contents). | 4 hours | In local venue (within walking distance of the homes of registered responders | Peer educators, bilingual general practitioner | *Not specified* |
| 29 | Islam et al 2013 | USA | Diabetes | Improving diabetes knowledge and self-management through a culturally and linguistically appropriate diabetes education intervention in a Bangladeshi population living in New York City. | The six-monthly group sessions were on topics: overview of diabetes, including myths and facts, disease specific information, and blood glucose levels; nutrition, physical activity, diabetes complications, stress and family support, and access to health care. And the 3 one-on-one visits during which challenges and strategies for diabetes management were discussed. | 6 monthly group sessions, then 3 one to one visit until 9 months | In clinical and community settings (home, community locations, restaurants) | Bilingual community health workers (who are community leaders) | *Not specified* |
| 30 | Trevisi et al, 2019 | USA | Diabetes | To improve self-management of diabetes and co-morbidities to reduce future cardiovascular risk through an intervention of diabetes education, participant coaching and strengthening the link between the health system and the community, in the Navajo community in the United States. | The “COPE Intervention” is comprised of three inter- related strategies designed to strengthen existing community outreach and linkage to clinic-based care: enabling patient referral to the Navajo Nation Community Health Representative Program, supporting community-based patient accompaniment, community-clinical linkages. | 2 years | Home visits | Community health representatives | *Not specified* |
| 31 | Spencer et al, 2018 | USA | Diabetes | To improve clinical parameters, diabetes self-management, understanding and knowledge of diabetes and psychosocial health status of people with diabetes through a diabetes education intervention among the Latino population in the United States. | The 6-month intervention, community health workers conducted: diabetes self-management education classes, two 60-min home visits each month, and one clinic visit with the participant and their primary care provider.  Peer leaders’ intervention was designed to provide patients with ongoing emotional and behavioral support through weekly drop-in group-based sessions and follow-up telephone contacts from 6 to 18 months. | 6 months, then 12 months follow-up | Community location and home visits | Community health worker and peer leader diabetes self-management | Anderson and Funnell’s empowerment conception |
| 32 | Beune et al,  2014 | The Netherlands | High blood pressure | Evaluate the effect of a practice-based, culturally appropriate patient education intervention on blood pressure (BP) and treatment adherence among patients of African origin with uncontrolled hypertension | Patients in the control sites received standard hypertension care and education  Patients at the intervention sites received usual care plus (i) three structured 30-minute culturally appropriate counselling sessions delivered by a trained practice nurse at 2 weeks, 8 weeks and at 20 weeks after baseline assessment; (ii) culturally appropriate written educational materials; and (iii) if applicable, referrals to neighborhood facilities, such as walking clubs and health food stores, that support patients in adopting healthier lifestyles and are suitable for Surinamese and Ghanaian people | 6 months follow-up | At primary care centers | A trained practice nurse | *Not specified* |
