# Supplementary material for: Empowerment-based support program for vulnerable populations living with diabetes, obesity or high blood pressure: a scoping review
Source: BMC Public Health. 2022 Nov 9;22:2051. doi: 10.1186/s12889-022-14480-3 (PMC9644395; doi:10.1186/s12889-022-14480-3)
Supplement: Supplementary file 3 — Additional file 3. [file 12889_2022_14480_MOESM3_ESM.docx]

**Appendix 3: Characteristics of included articles and interventions (continued)**

| **Reference** | **Author and year of publication** | **Study design** | **Type of data collected** | **Indicators** | **Sample size** | **Main results** |
| --- | --- | --- | --- | --- | --- | --- |
| 17 | Hill et al, 2011 | Randomized controlled trial | Quantitative data | Clinical measures (HbA1c, LDL and HDL, blood pressure). Knowledge Diabetes and cardiovascular diseases Knowledge Test. Problem-solving (Health Problem-Solving Scale (HPSS)). Behaviors (Summary of Diabetes Self-Care Activities scale (SDSCA)). Barriers (item from the Michigan Diabetes Training and Research Center Diabetes History Questionnaire). Patient Satisfaction and Usability | 56 | The traditional PST delivery model (intensive), but not the abbreviated model is effective for key diabetes behavioral and clinical outcomes. At 3 months post-intervention, only the intensive intervention was effective in improving knowledge, problem-solving skills, self-care, and A1C. At immediate post-intervention, participants in both programs demonstrated knowledge gain. Participants in each intervention experienced the program as helpful and easy to understand |
| 18 | Tucker et al, 2014 | Stratified random sampling approach | Quantitative data | Clinical measures (BMI, systolic and diastolic blood pressure, self- reported levels of blood glucose). Treatment adherence. Overall stress, physical stress, cognitive stress, and behavioral stress | 130 | The Intervention Group evidenced significantly lower levels of diastolic blood pressure and physical stress at the 2-month post program data collection as compared to the participants in the Control Group and a significant decrease in BMI of 0,5 or greater. |
| 19 | Tang et al, 2012 | Single cohort, longitudinal prospective study | Quantitative data | Clinical measures (HbA1C, Lipids, Blood pressure, weight, BMI). Self-care behaviors (Summary of Diabetes Self-Care Activities Measure-revised). Psychosocial indices (QoL: Diabetes Distress Scale (DDS), Empowerment: Diabetes Empowerment Scale-short Form) | 52 | Results at 24 months (post-intervention): significant improvements for following a healthy diet, spacing carbohydrates evenly across the day, using insulin as recommended, and achieving diabetes-specific quality of life  Results at 36 months (1 year follow-up): participants sustain the behavioral improvements made in the 2-year diabetes self-management support intervention, additional improvements in glycemic control, and in serum cholesterol and low-density lipoprotein cholesterol levels |
| 20 | Peek et al, 2012 | Observational cohort study | Quantitative data | Clinical outcomes (HbA1c, systolic blood pressure, diastolic blood pressure, cholesterol (LDL and HDL), weight, and BMI). Diabetes self-efficacy. Diabetes self-care measures. Shared decision-making self-efficacy. Shared decision-making behavioral measures. Patient satisfaction and program evaluation. | 21 | There were improvements in diabetes self-efficacy, self-care behaviors, self-glucose monitoring, and foot care, hemoglobin A1c, and HDL cholesterol. Combining tailored education with shared decision-making may be a promising strategy for empowering low-income African-Americans and improving health outcomes. |
| 21 | Tucker et al, 2016 | Comparative study | Quantitative data | Clinical outcomes (height, weight, and diastolic and systolic blood pressure). Health-Promoting Lifestyles Profile. Motivators of and Barriers to Health-Smart Behaviors Inventory. | 70 | Both the intervention group and the waitlist control group experienced improvements in their levels of engagement in healthy eating and physical activity from pre-intervention to post-intervention. Intervention group experienced a greater increase in level of engagement in healthy eating compared to the waitlist control group and a greater increase in levels of engagement in physical activity compared to the waitlist control group. Neither group experienced significant changes in BMI, the intervention group experienced a statistically significant decrease in weight compared to the waitlist control group, and neither group experienced significant changes in systolic and diastolic blood pressure. |
| 22 | Fernandes et al, 2017 | Observational study, a randomized controlled trial (RCT) and a cost analysis | Quantitative data | Clinical outcomes (weight, height, body mass index, blood pressure, fasting blood glucose, hemoglobin A1c, fasting lipid profile, renal function, smoking cessation, retinopathy, and influenza/pneumococcal vaccination status) | 2003 in the observational study and 320 in the  RCT | Observational study: The project showed statistical improvements in key clinical outcomes of HbA1c, blood pressure, and cholesterol along with increased participant compliance with American Diabetes Association standards of diabetes care in the observational study.  RCT: No statistically significant improvements in the clinical measures were observed  Cost analysis: The project did not show reduction in health cost at the end of the study |
| 23 | Anderson et al, 2005 | Randomized controlled trial | Quantitative data | Clinical outcomes (HbA1C, lipids, blood pressure, weight). Diabetes Care Profile. The Diabetes Empowerment Scale Short-Form. The ‘‘Seriousness of Diabetes’’ subscale of the Diabetes Attitude Scale-3. | 239 | Both control and intervention patients showed a broad array of small-to-modest positive changes during the six-week RCT. These gains were maintained or improved upon during the one-year follow-up period. For patients in the two follow-up conditions, a positive correlation was seen between the number of follow-up contacts and their one- year HbA1C values. |
| 24 | Sorkin et al, 2014 | Randomized controlled trial | Quantitative data | Clinical outcomes (weight). Health-related social support, social control (persuasion and pressure) and undermining. Detailed assessment of dietary intake. | 178 (89 dyads mother-daughter) | The Unidas program promoted weight loss and improved dietary intake, as well as changes in diet-related involvement of participants’ social networks. |
| 25 | Hawthorne, 2001 | Randomized controlled trial | Quantitative data | Clinical outcomes (HbA1c, diet, diabetic complications). Knowledge score and reasons for the management diabetes) | 105 | Nearly everyone improved their knowledge scores after 6 months in the intervention group, with women showing a catch-up improvement such that they equaled men. Multiple regression analysis found that glycemic control improved in women receiving health education. Although this method of health education improved knowledge and glycemic control in women in this sample, illiterate women did not do as well as their literate peers, continuing to score less on knowledge parameters. They also did not show an improvement in glycemic control. |
| 26 | Vyas et al, 2003 | Randomized controlled trial | Quantitative and qualitative data | Knowledge and awareness. Self-management scores. | 118 | Intervention had no impact on scores for diabetes knowledge, or awareness or self-management between baseline and 1 year.  This form of secondary/primary care support did not transfer information effectively, and we suspect similar problems would arise in other similar communities. Most patients felt that their diabetes was a significant burden in their physical and social life. |
| 27 | Bellary et al, 2008 | Cluster randomized controlled trial and a cost analysis | Quantitative data | Clinicals outcomes (blood pressure, total cholesterol, and glycemic control (HbA1c)) | 1486 | Significant differences between treatment groups in diastolic blood pressure and mean arterial pressure, after adjustment for confounders and clustering.  No significant differences between groups for total cholesterol, systolic blood pressure, or HbA1c  And the economic analysis suggests that the nurse-led intervention was not cost effective.  Across the whole study population over the 2 years of the trial, systolic blood pressure, diastolic blood pressure, and cholesterol decreased significantly and we recorded a small and non-significant increase for hemoglobin A1c. |
| 28 | Choudhury et al, 2008 | Observational study | Quantitative and qualitative data | Summary of Diabetes Self-Care Activities (SDSCA) questionnaire. Monitoring data (number of people attending the course, number registered to attend). Satisfaction. | 42 | Registration to attend the course was excellent.  Once participants attended, overall, they enjoyed attending the sessions and felt they benefited as they understood more about how to better self-manage their condition. Those aspects of the sessions that were most enjoyed were group discussions and interactive posters to explain diabetes. Participants requested a home-based exercise guide, which was developed by the research team. The study was underpowered to show behavior change, but did demonstrate a trend to improvement in self-care activities. |
| 29 | Islam et al 2013 | Before and after study | Quantitative and qualitative data | Clinical outcomes (HbA1c, weight, nutritional and physical activity behaviors, and access to healthcare). Diabetes knowledge scale. Diabetes management and knowledge. Medication. Self-efficacy questions on nutrition and physical activity, and the self-efficacy health access scale. Mental health questions. Participant satisfaction | 26 | Improvements were seen in diabetes knowledge, exercise and diet to control diabetes, frequency of checking feet, medication compliance, and self-efficacy of health and physical activity from baseline to 12 months and a decrease in A1C, weight, and BMI. |
| 30 | Trevisi et al, 2019 | Quasi-experimental study (intervention vs control) | Quantitative data | Clinical outcomes (HbA1c, low-density lipoprotein, systolic blood pressure and  body mass index). | 3053 | Participation in COPE among individuals living with T2DM was associated with improvements in HbA1c at 12 and 24 months and in LDL at 24 months: a clinically meaningful change which surpassed the average responses observed in T2DM self-management education programs described in a recent meta- analysis; a significant reduction in LDL among COPE patients compared to non-COPE patients; a slight increase in blood pressure for both COPE and non-COPE patients. We did not see improved changes in body mass index, or monitoring of standard clinical measures related to cardiovascular risk due to COPE participation. |
| 31 | Spencer et al, 2018 | Randomized controlled trial | Quantitative data | Clinical outcomes (blood pressure, lipid levels, weight, height and waist circumference, BMI). Diabetes distress. Depressive symptoms. Understanding of diabetes self-management.  Diabetes-specific social support. | 222 | Participants in the intervention at the 6-month follow-up had greater decreases in HbA1c and in diabetes distress compared with control. Participants who received the intervention and peer support had significant improvements at 12 and 18 months, and participants who received the intervention only maintained improvements in diabetes-related distress at 12 and 18 months. Participants who received the intervention and peer support also had significantly fewer depressive symptoms at 18 months compared with the control group. Participants who received the intervention showed significant improvements in diabetes social support and understanding of diabetes self-management at 6 months compared with the control group, but these intervention effects were not maintained at 18 months. |
| 32 | Beune et al,  2014 | Cluster randomized trial | Quantitative data | The primary outcome was the between-group difference in the proportion of patients with a SBP reduction of at least 10 mmHg at 6 months. The secondary outcomes were the mean between- group differences in changes in SBP and DBP and adherence to lifestyle and medication recommendations from baseline to 6 months | 146 | A systolic BP reduction of 10 mmHg or more in 48% of the patients in the intervention group and 43% in the control group, but the between-group difference was not statistically significant.  The mean between-group differences (intervention and control) for systolic and diastolic BP reduction were 21.7 mmHg and 23mmHg respectively, and statistically significant for the diastolic BP (P = 0.03).  The intervention was associated with a significant improvement of self-reported adherence to lifestyle recommendations |
