# Supplementary material for: Empowerment-based support program for vulnerable populations living with diabetes, obesity or high blood pressure: a scoping review
Source: BMC Public Health. 2022 Nov 9;22:2051. doi: 10.1186/s12889-022-14480-3 (PMC9644395; doi:10.1186/s12889-022-14480-3)
Supplement: Supplementary file 4 — Additional file 4. [file 12889_2022_14480_MOESM4_ESM.docx]

**Appendix 4: Detailed description of intervention characteristics**
